# Population Structure of Mixed *Mycobacterium tuberculosis* Infection Is Strain Genotype and Culture Medium Dependent

**DOI:** 10.1371/journal.pone.0070178

**Published:** 2013-07-30

**Authors:** Madeleine Hanekom, Elizabeth M. Streicher, Doreen Van de Berg, Helen Cox, Cheryl McDermid, Marlein Bosman, Nicolaas C. Gey van Pittius, Tommie C. Victor, Martin Kidd, Dick van Soolingen, Paul D. van Helden, Robin M. Warren

**Affiliations:** 1 Division of Anatomy and Histology, Faculty of Health Sciences, Tygerberg, Stellenbosch University, Stellenbosch, Western Cape, South Africa; 2 DST/NRF Centre of Excellence for Biomedical Tuberculosis Research/MRC Centre for Molecular and Cellular Biology, Division of Molecular Biology and Human Genetics, Faculty of Health Sciences, Tygerberg, Stellenbosch University, Stellenbosch, Western Cape, South Africa; 3 Centre for Infectious Disease Epidemiology and Research, Cape Town University, Cape Town, South Africa; 4 Médecins Sans Frontières, Cape Town, South Africa; 5 National Health Laboratory Service, Cape Town, Western Cape, South Africa; 6 Centre for Statistical Consultation, Stellenbosch University, Stellenbosch, Western Cape, South Africa; 7 National Tuberculosis Reference Laboratory, National Institute for Public health and the Environment (RIVM), Bilthoven, The Netherlands; McGill University, Canada

## Abstract

**Background:**

Molecular genotyping methods have shown infection with more than one *Mycobacterium tuberculosis* strain genotype in a single sputum culture, indicating mixed infection.

**Aim:**

This study aimed to develop a PCR-based genotyping tool to determine the population structure of *M. tuberculosis* strain genotypes in primary Mycobacterial Growth Indicator Tubes (MGIT) and Löwenstein–Jensen (LJ) cultures to identify mixed infections and to establish whether the growth media influenced the recovery of certain strain genotypes.

**Method:**

A convenience sample of 206 paired MGIT and LJ *M. tuberculosis* cultures from pulmonary tuberculosis patients resident in Khayelitsha, South Africa were genotyped using an in-house PCR-based method to detect defined *M. tuberculosis* strain genotypes.

**Results:**

The sensitivity and specificity of the PCR-based method for detecting Beijing, Haarlem, S-family, and LAM genotypes was 100%, and 75% and 50% for detecting the Low Copy Clade, respectively. Thirty-one (15%) of the 206 cases showed the presence of more than one M. tuberculosis strain genotype. Strains of the Beijing and Haarlem genotypes were significantly more associated with a mixed infection (on both media) when compared to infections with a single strain (Beijing MGIT p = 0.02; LJ, p<0.01) and (Haarlem: MGIT p<0.01; LJ, p = 0.01). Strains with the Beijing genotype were less likely to be with “other genotype” strains (p<0.01) while LAM, Haarlem, S-family and LCC occurred independently with the Beijing genotype.

**Conclusion:**

The PCR-based method was able to identify mixed infection in at least 15% of the cases. LJ media was more sensitive in detecting mixed infections than MGIT media, implying that the growth characteristics of *M. tuberculosis* on different media may influence our ability to detect mixed infections. The Beijing and Haarlem genotypes were more likely to occur in a mixed infection than any of the other genotypes tested suggesting pathogen-pathogen compatibility.

## Introduction

The risk of a subsequent episode of disease after cure due to a new pulmonary tuberculosis (TB) infection, usually referred to as exogenous reinfection, is about 4–7 times greater than the risk of a first-time infection that leads to disease [1], [2]. This is especially true in settings where the infection pressure is high, like in South Africa [3]. More recently, advanced molecular epidemiological tools have shown infection with more than one strain during the same episode (mixed infection) [3]–[6].

Using molecular genotyping methods, the frequency of mixed infection was shown to range from 2.8% to 19% in countries like Malawi, China, Uganda, Taiwan, Georgia, Central Asia and South Africa [3],[5]–[10]. From these studies it was concluded that mixed infections could influence the diagnosis of drug resistance if a patient is infected with both a drug sensitive and drug resistant strain [11]. In such instances the patient may either be exogenously reinfected with a drug resistant strain while on treatment for drug susceptible TB [12] or the patient may have been infected with both a drug sensitive and drug resistant strain prior to progression of disease. In the latter case, subsequent treatment would select for the drug resistant strain leading to drug resistant TB [12], [13]. Thus mixed infection could influence treatment outcome. Furthermore, it has also been hypothesized that infection with a second strain may reactivate the primary infection [14].

The true extent of mixed infections remains unclear, since detection is limited to the sensitivity of the genotyping method [4], the culture media used [15], [16] or spread of infection to multiple anatomical sites which are not similarly connected to the airway [17], [18]. Currently it is not known how delays and variation in culture, as well as the decontamination process could influence the identification of mixed infections.

To date, no studies have been done to determine whether certain strains are more likely to cause mixed infections and whether the media influences the population structure of *M. tuberculosis* in mixed infections. To test this hypothesis, we adapted the PCR method of Mallard *et al* (2010) to enhance the discriminatory power to detect the presence or absence of 5 different *M. tuberculosis* strain genotypes (Beijing, LAM, S-family, Low Copy Clade (LCC) and Haarlem) [7]. This method was then applied to identify mixed infections in primary cultures grown on different media (Mycobacteria growth incubator tube (MGIT) and Löwenstein-Jensen (LJ) media) to determine the influence of the culture media on the growth of different strains.

## Methods

### Ethics Statement

The survey study was explained to clinic attendees who were eligible to participate and written informed consent was obtained from each participant. The survey was approved by the University of Cape Town Ethical Review Committee and by both the City of Cape Town and the Western Cape Province Health department. This study was approved by the Health Research Ethics Committee of Stellenbosch University.

### Study Population

A cross-sectional survey among clinic attendees with presumptive pulmonary TB (PTB) was conducted in 2 large primary care clinics in Khayelitsha between May and November 2008 [19]. Sputum culture isolates were available from 535 TB patients identified through the survey. In Khayelitsha, South Africa, the case notification of TB is reported to be at least 1500/100 000 per annum and the antenatal HIV infection rate is 33% (City of Cape Town, Health Department, Statistics. 2009).

All sputum specimens were submitted to the National Health Laboratory Service for culture and drug susceptibility testing (DST). After decontamination, each sputum specimen was cultured in MGIT liquid medium (Becton-Dickinson) and on LJ solid medium. Aliquots of the respective primary cultures were boiled at 100°C for 30 min to ensure sterilization and release of the *M. tuberculosis* DNA.

### PCR Detection of *M. tuberculosis* Strain Genotypes

To determine the presence of a particular *M. tuberculosis* strain genotype in the MGIT and LJ cultures, a boiled aliquot of each culture was PCR amplified as previously described [3], using the primers described in Table 1. PCR amplification products were electrophoretically fractionated in 2.0% agarose in 27 mM di-sodium tetraborate buffer (SB) pH 8.3 at 3.5 V/cm for 4 hours and visualized by staining with ethidium bromide. The presence of a particular *M. tuberculosis* strain genotype was defined when the amplified product corresponded to the predicted product size (Table 1). An “other genotype” was assigned if all of the amplified regions gave product sizes which could not be assigned to a defined genotype using the 5 primer sets. The presence of DNA from two or more *M. tuberculosis* strain genotypes in a culture was classified as a mixed infection.

**Table 1:** Primer sequences used to identify strains of the Beijing, LAM, S-family, LCC and Haarlem genotype, respectively.

Primerset	Primer	Primer sequence (5′ –3′)	PCR product 1(base pairs)	Genotype	PCR product 2(base pairs)	Genotype
1	RD105^b^ F	ACA GCG CGG GTC ATA TCA C	405	Beijing	615	non-Bejing
	RD105 R	AAC CAG CTC CTC GAC GCT ATC				
	RD105 INT	GCA ACA CCC GCT TGT CTT TG				
2^a^	Lam F	TAG CCC ACC ACC ACA GCT TC	205	LAM	141	non-LAM
	Lam R	ACC ACC CTG CCT AAC CAA TTC				
3	F28/480.9 F	GGC GGT GTT AGC GAT TGA A	194	S	236	non-S
	F28/480.9 R	CTG CGG CAA CAG ATT CCA CTA				
4	Rv0403c F	GAC AAC GCA TGG ATC GTC C	270	LCC	388	non-LCC
	Rv0403c R	TCA CAT CAA CAT GCG CCC				
5	Rv2336 F	GGT GGC GAA AGC TTT AGC C	279	Haarlem	212	non-Haarlem
	Rv2336 R	TGC GCC AAA CAT GCA GTC				
	Internal primer^c^	TTC AAC CAT CGC CGC CTC TAC				

a according to [20].

b RD105 region according to [22].

c internal primer used for primer set 2 to 4.

To minimize the risk of laboratory cross-contamination during the PCR amplification, each procedure (preparation of the PCR reaction mixes, the addition of the DNA, the PCR amplification and the electrophoretic fractionation) was done in physically separated rooms. Negative controls (water) were included to monitor for reagent contamination.

The sensitivity and specificity of amplification for the different primer sets was determined by PCR amplification of a panel of genetically related and unrelated *M. tuberculosis* strains classified according to IS*6110* RFLP and spoligotyping [3]. Briefly, DNA from 40 genetically known related and unrelated *M. tuberculosis* strains, representing 31 distinct strains, were amplified with primer sets 1 to 5.

### Statistical Methods

The Kappa coefficient of concordance was used as a measure of concordance between the MGIT and LJ cultures. Comparisons of binary responses were done using cross tabulation and the Chi-square test.

Data may be accessed on request from the corresponding author.

## Results

### Study Population

Among the 535 culture positive patients identified through the drug resistance surveillance survey, a convenience sample of 206 paired MGIT and LJ cultures were available for subsequent analysis. The patients represented in this cohort were not statistically different from the patients in the entire survey cohort [19], with the exception of previous TB treatment resulting from extended sampling of individuals with presumptive TB in this category in the original survey.

### PCR Detection of *M. tuberculosis* Strain Genotypes

A simple PCR method was designed to detect the presence of five distinct *M. tuberculosis* strain genotypes. Primers were designed (Table 1), complementary to the internal sequence of IS*6110* and the 3′ and 5′ insertion sequence junctions of *M. tuberculosis* strains to identify different genotypes, as previously described [12]. Only primer sets that uniquely amplified strains representative of the LAM [20], S, LCC and Haarlem genotypes [21] were selected for subsequent analysis (Table 1). In addition, a primer set spanning the junction site of the Region of Difference (RD) 105 was used to specifically identify the presence of Beijing genotype strains (Table 1) [22].

The sensitivity and specificity of primer set 1, 2, 3 and 5 for detecting the Beijing, LAM, S and Haarlem genotypes was 100% (95%CI 85–100%), respectively, while the sensitivity of primer set 4 for detecting the LCC genotype was 75% and specificity 50% (95%CI 85–100%) as this primer set recognized *M. tuberculosis* strains in both the LCC and Haarlem genotypes.

### Detection of Mixed Infection

The PCR-based method identified 180 (87.4) and 174 (84.5%) single infections on MGIT and LJ media, respectively among the 206 isolates (Table 2). Concordance between the MGIT and LJ cultures showed a Kappa value of 0.75 (0.61–0.88). The PCR-based method identified 23 mixed infections on MGIT media and 28 on LJ media. In combination, 31 (15%) mixed infections were identified by culture on both MGIT and LJ media. The population structure of these mixed infections included 20 Beijing/non-Beijing strain genotype combinations and 11 non-Beijing genotype combinations (Table 3).

**Table 2:** Strain genotypes cultured on MGIT and LJ media classified according to PCR amplification.

		MGIT media		LJ media
Category	Strain genotypes [21]	Cases (%)	Cases (%)	
		n = 206	n = 206	
Single infections		180 (87.4)	174 (84.5)	
	Beijing	63 (30.6)	53 (25.7)	
	LAM	64 (31.1)	66 (32.0)	
	S-family	13 (6.3)	13 (6.3)	
	LCC	19 (9.2)	19 (9.2)	
	Haarlem	3 (1.5)	4 (1.9)	
	“Other genotype”^a^	18 (8.7)	19 (9.2)	
No amplification^b^		3 (1.5)	3 (1.5)	
Unknown product^b^		0	1 (0.5)	
Mixed infections^c^		23 (11.1)^d^	28 (13.6)^d^	

a strain genotypes other than the strains tested with the 5 primer sets.

b isolates did not amplify or amplified with an unknown product.

c more than 1 strain genotype was present in the primary culture.

d in combination, mixed infection was observed in 15% of cases.

**Table 3:** The population structure of mixed infections cultured on MGIT media and their corresponding LJ media.

	MGIT media	LJ media
1	Beijing+LAM	Beijing+LAM
2	Beijing+LAM	Beijing+LAM
3	Beijing+LAM	Beijing+LAM
4	Beijing+LAM	Beijing+LAM
5	Beijing+LAM	Beijing+LAM
6	Beijing+LAM	Beijing+LAM
7	Beijing+LAM+LCC	Beijing+LAM
8*	Beijing	Beijing+LAM
9*	Beijing	Beijing+LAM
10*	Beijing	Beijing+LAM
11*	Beijing	LAM+“Other genotype”
12	Beijing+LCC	Beijing+LCC
13	Beijing+LCC	Beijing+LCC
14*	Beijing	Beijing+LCC
15	Beijing+Haarlem	Beijing+Haarlem
16	Beijing+Haarlem	Beijing+Haarlem
17	Beijing+Haarlem	Beijing+Haarlem
18	Beijing+“Other genotype”	Beijing+LAM+S-family+LCC
19*	Beijing	Beijing+LAM+S-family+LCC
20*	Beijing	Beijing+“Other genotype”
21	Beijing+S-family	Beijing+S-family
22	LAM+“Other genotype”	LAM+“Other genotype”
23*	LAM+“Other genotype”	LAM
24*	LAM+“Other genotype”	LAM
25	LCC+“Other genotype”	LCC+“Other genotype”
26	LCC+“Other genotype”	LCC+“Other genotype”
27	S-family+“Other genotype”	S-family+“Other genotype”
28*	Haarlem+“Other genotype”	Haarlem
29	LAM+LCC	LAM+LCC
30*	LAM	LAM+LCC
31	LAM+Haarlem	LAM+Haarlem

* discrepant PCR amplification results of strain genotypes cultured on MGIT or LJ media, respectively.

### Effect of Culture Media

Twenty (64.5%) of the mixed infections showed concordant strain genotypes on both MGIT and LJ media (an additional strain genotype was identified on one of the cultures grown on MGIT media – case #3) (Table 3). Furthermore, 10 (32.3%) of the mixed infections showed that one of the strain genotypes was present on both media while the other strain genotype was present on either the MGIT or LJ media (Table 3). The remaining mixed infection (3.2%) showed dissimilar strain genotypes on both media (Table 3).

Strains of the Beijing (p = 0.02) and Haarlem (p<0.01) genotypes were significantly more likely to be in a mixed infection when grown on MGIT and LJ media while strains with the “other genotype” (p<0.01) were significantly more likely to be in a mixed infection when grown on MGIT media (Table 4). LAM (p = 0.02) and LCC (p<0.01) genotypes were significantly associated with mixed infections when grown on LJ media (Table 4). Strains with the Beijing genotype were less likely to be with “other genotype” strains (p<0.01) while LAM, Haarlem, S-family and LCC occurred independently with the Beijing genotype in mixed infections cultured on either MGIT or LJ media (Table 5).

**Table 4:** Strain genotypes present in single and mixed infections.

	MGIT media			LJ media		
Strain genotypes[21]	Single infections	Mixed infections	p-value	Single infections	Mixed infections	p-value
	n = 180	n = 23		n = 174	n = 28	
Beijing	63	14	0.02	53	20	<0.01
Haarlem	3	5	<0.01	4	4	0.01
LAM	64	12	0.11	66	17	0.02
LCC	19	6	0.05	19	9	<0.01
S-family	13	2	0.79	13	4	0.25
“Other genotype”	18	8	<0.01	19	6	0.13

**Table 5:** Frequency of strain genotype combinations cultured on MGIT or LJ media.

	MGIT media			LJ media		
Genotype present	Beijing present	Beijing absent	p-value	Beijing present	Beijing absent	p-value
	n = 14 (%)	n = 9 (%)		n = 20 (%)	n = 8 (%)	
LAM	7 (50)	5 (56)	0.79	12 (60)	5 (63)	0.90
Haarlem	3 (21)	2 (22)	0.96	3 (15)	1 (13)	0.86
LCC	3 (21)	3 (33)	0.52	5 (25)	4 (50)	0.20
S-family	1 (7)	1 (11)	0.74	3 (15)	1 (13)	0.86
“Other genotype”	1 (7)	7 (78)	<0.01	1 (5)	5 (63)	0.01

Two of the 10 patients with isoniazid (INH) mono-resistant TB were infected with Beijing and LAM genotype strains which were present on both MGIT and LJ media. One patient, who was infected with a fully susceptible isolate at baseline and developed MDR-TB by month 3 of treatment, showed the presence of Beijing and LAM genotype strains on LJ media at baseline.

## Discussion

In this study a PCR-based method was developed to describe the population structure of *M. tuberculosis* strain genotypes in primary cultures from patients diagnosed with pulmonary TB who were resident in a high TB/HIV setting in South Africa. Using this method, it was possible to identify members of the Beijing, LAM, S-family, LCC and Haarlem genotypes. A previous study showed that using this approach it was possible to detect underlying strains at a proportion of 1∶125, making it highly sensitive for the detection of mixed infections [12]. However, it is acknowledged that our method may not be able to accurately identify mixed infections when the relative proportion of two different strain genotypes is greater than 1∶125. Furthermore, our method was limited by the fact that it could only detect the presence or absence of a defined strain genotype and thus it was not possible to determine the extent of mixed infection with different strains from the same genotype. Together these limitations could lead to an under-estimate of the frequency of mixed infection thereby clouding their epidemiological importance. The true proportion of mixed infection can only be accurately quantified by methods that discriminate at a strain level [10].

Despite these limitations we were able to demonstrate that at least 15% of sputum cultures from individual TB cases contained *M. tuberculosis* DNA from more than one strain genotype. This was lower than the frequency of mixed infections described by using a PCR-based method which could only differentiate between Beijing and non-Beijing genotypes [3]. This may be explained by the characteristics of the two study settings; in this study setting the proportion of smear positive cases was lower (55% vs. 94%), while the HIV/TB co-infection rate was higher (56% vs. ±10%) than that reported by Warren *et al* (2004) [3]. Furthermore, it is well established that HIV positive individuals are at a greater risk of rapidly progressing to disease following infection [23], thus their risk of being reinfected during the period prior to the onset of disease would be reduced.

We cannot exclude the possibility that laboratory cross-contamination could have contributed to a proportion of the observed mixed infections. However, a previous analysis of the laboratory where these isolates were cultured estimated cross-contamination to be of the order of 3.8% [3].

Our PCR-based method provided insight into the *M. tuberculosis* strain population structure in sputum cultures. We showed that the majority of mixed infection contained two distinct strain genotypes, however, in some instances four different genotypes were present in a single patient. This is similar to previous studies which have reported three to four different strains being present [24], [25]. In addition, we showed that strains of the Beijing genotype were over-represented in mixed infections of cultures grown on either MGIT or LJ media. In mixed infections, strains of the Beijing genotype occurred in combination with all other genotypes tested (LAM, LCC, “other genotype”, S-family, Haarlem), however, Beijing genotype strains were less likely to occur with strains with the “other genotype”. LAM, Haarlem, S-family and LCC occurred independently with the Beijing genotype in mixed infections cultured on either MGIT or LJ media. We hypothesize that this may reflect pathogen-pathogen compatibility within the human host, whereby the Beijing genotype has either evolved properties allowing it to reinfect patients more readily or conversely patients already infected with a Beijing genotype strain may be more vulnerable to reinfection with certain non-Beijing strains. However, due to the small sample size further studies are needed to determine whether certain genotypes influence the growth of other genotypes *in vivo* and whether certain strain genotypes influence the rate of disease progression.

In this study, the population structure of mixed *M. tuberculosis* strain genotypes was concordant in 64.5% of the cultures when cultured on either MGIT or LJ media. Discordance where either one or both of the strain genotypes were absent from either the MGIT or LJ media was identified in 31.8% of the cultures, while unrelated strain genotypes were identified on both media in 3.2% of the cultures. Twenty-six percent of the mixed infections were not detected by culture on MGIT media, while 10% were not detected when cultured on LJ media. Our observations support previous reports which have suggested the use of a combination of both liquid and culture media for maximum recovery of *M. tuberculosis* [15], [26], thereby avoiding a “microbiological bias” [15], [26].

In summary, this study describes the use of a PCR-based genotyping method to identify distinct *M. tuberculosis* strain genotypes in primary cultures. Using this method we showed that TB cases may be infected with more than one strain genotype. Analysis of the population structure of *M. tuberculosis* strain genotypes showed that certain strain genotypes were over-represented in mixed infections and that the growth characteristics of *M. tuberculosis* on different media may influence our ability to detect mixed infections.
